# Complete genome sequence of *Veillonella parvula* strain PK1910

**DOI:** 10.1128/mra.00761-24

**Published:** 2025-02-25

**Authors:** Allison A. Naumann, Danyal Siddiqui, Kristopher A. Kerns, Erik L. Hendrickson, Georgios A. Kotsakis, Jeffrey S. McLean

**Affiliations:** 1 Department of Periodontics, University of Washington, Seattle, Washington, USA; 2 Department of Oral Biology & Clinical Research Center, Rutgers School of Dental Medicine, Newark, New Jersey, USA; 3 Department of Microbiology, University of Washington, Seattle, Washington, USA; 4 Department of Oral Health Sciences, University of Washington, Seattle, Washington, USA; Loyola University Chicago, Chicago, Illinois, USA

**Keywords:** genome sequence, periodontitis, peri-implantitis, *Veillonella*

## Abstract

Here, we announce the complete genome sequence of *Veillonella parvula* strain PK1910, obtained from a frozen stock. The genome is composed of one closed contig with a length of 2,213,486 bp, resulting in 98.0× coverage containing 1,979 protein-coding genes, with a GC content of 38.74%.

## ANNOUNCEMENT

Bacteria within *Veillonella* are common members of the oral microbiome, residing in biofilms on both supra- and subgingival plaque, tongue, and buccal mucosa. Members of *Veillonella* are significant contributors to periodontal diseases, including peri-implantitis (1). Here, we present the complete genome sequence of *Vp* strain PK1910, isolated from subgingival plaque samples (2). PK1910 is the first *Veillonella* strain to have a genetic system developed (3). This completed genome can assist in analyzing the relationship between *Veillonella parvula* strains and other species. It provides a reference genome for microbiome studies and allows for examination of this strain’s impact on the oral microbiome and subsequential oral health.

Strain PK1910, donated by Dr. Peng Zhou, was grown on BHI agar supplemented with 0.6% (vol/vol) sodium DL-lactate and incubated for 24–48 hours at 37°C under anaerobic conditions. Colonies were inoculated in 5 mL of BHI broth supplemented with sodium DL-lactate, porcine hemin, and vitamin K1 and incubated under anaerobic conditions for 10.5 hours. Culture quality was determined by optical density, which resulted in an OD_600_ of 0.948. DNA was extracted by mechanical and chemical lysis, followed by inhibitor removal and elution using the Qiagen QIAmp PowerFecal Pro DNA Kit. The quality of gDNA was determined by Qubit 4 Fluorometer, giving a sample concentration of 426 ng/µL. Hybrid bacterial sequencing was performed by Plasmidsaurus using Oxford Nanopore Technology with custom analysis and annotation, as described below. ONT and Illumina sequencing were run on PromethION P24 and NextSeq 2000, respectively.

For *de novo* assembly, the bottom 5% of worst fastq reads were removed via Filtlong version 0.2.1 (https://github.com/rrwick/Filtlong) using default parameters. The reads were downsampled to 250 Mb to create a rough sketch of the assembly with Miniasm version 0.3 (4). The reads were downsampled to approximately 100× coverage with weight applied to remove low-quality reads. A Flye version 2.9.1 (5) assembly was run with parameters intended for high-quality ONT reads, then polished via Medaka version 1.8.0 using the re-downsampled reads. Annotation was completed with Bakta version 1.6.1. Contig analysis ran with Bandage version 0.8.1 resulted in a singular, closed contig. Genome completeness was checked with CheckM version 1.2.2 resulting in 100% completeness with 0% contamination. The consensus .fna was copied from the ONT-only assembly. BWA was used to align raw Illumina .fastq to the ONT-only assembly (6). The .sam alignment file was used as input for Polypolish version 0.5.0 (7), which yielded the completed singular contig and annotated with NCBI Prokaryotic Genome Annotation Pipeline (version 6.6 August 2023). The completed genome is 2,213,486 bp with a G + C content of 38.74%. Annotations described above identified a total number of 2,049 genes, with 1,979 coding sequences, 48 tRNAs, 4 5S rRNAs, 4 large-subunit 23s rRNAs, and 4 small-subunit 16S rRNAs.

## Data Availability

This genome project is indexed at GenBank under BioProject accession number PRJNA1039853. The complete genome sequence for *Veillonella parvula* PK1910 is under GenBank accession number GCA_036456085.1. Raw reads are available in the Sequence Read Archive (SRA) under submission numbers SRR29735640 and SRR29735641 for the Oxford Nanopore and Illumina reads, respectively.
